# Co-culture of Retinal and Endothelial Cells Results in the Modulation of Genes Critical to Retinal Neovascularization

**DOI:** 10.1186/2045-824X-3-27

**Published:** 2011-11-23

**Authors:** Ravindra Kumar, Sandra Harris-Hooker, Ritesh Kumar, Gary Sanford

**Affiliations:** 1 Department of Microbiology, Biochemistry and Immunology, Morehouse School of Medicine, 720 Westview Drive, S.W., Atlanta, Georgia, United States 30310; 2 Department of Pathology, Morehouse School of Medicine, 720 Westview Drive, S.W., Atlanta, Georgia, United States 30310; 3 Undergraduate student, Department of Chemistry and Biochemistry, Georgia Institute of Technology, 901 Atlantic Drive, Atlanta Georgia, United States 30322

**Keywords:** Neovascularization, Human retinal progenitor cells (HRPC), Human umbilical vein endothelial cells (HUVEC), Hypoxia, Vascular endothelial growth factor, Conditioned medium, Co-culture

## Abstract

**Background:**

Neovascularization (angiogenesis) is a multistep process, controlled by opposing regulatory factors, which plays a crucial role in several ocular diseases. It often results in vitreous hemorrhage, retinal detachment, neovascularization glaucoma and subsequent vision loss. Hypoxia is considered to be one of the key factors to trigger angiogenesis by inducing angiogenic factors (like VEGF) and their receptors mediated by hypoxia inducible factor-1 (HIF-1α) a critical transcriptional factor. Another factor, nuclear factor kappa B (NFκB) also regulates many of the genes required for neovascularization, and can also be activated by hypoxia. The aim of this study was to elucidate the mechanism of interaction between HRPC and HUVEC that modulates a neovascularization response.

**Methods:**

Human retinal progenitor cells (HRPC) and human umbilical vein endothelial cells (HUVEC) were cultured/co-cultured under normoxia (control) (20% O_2_) or hypoxia (1% O_2_) condition for 24 hr. Controls were monolayer cultures of each cell type maintained alone. We examined the secretion of VEGF by ELISA and influence of conditioned media on blood vessel growth (capillary-like structures) via an angiogenesis assay. Total RNA and protein were extracted from the HRPC and HUVEC (cultured and co-cultured) and analyzed for the expression of VEGF, VEGFR-2, NFκB and HIF-1α by RT-PCR and Western blotting. The cellular localization of NFκB and HIF-1α were studied by immunofluorescence and Western blotting.

**Results:**

We found that hypoxia increased exogenous VEGF expression 4-fold in HRPC with a further 2-fold increase when cultured with HUVEC. Additionally, we found that hypoxia induced the expression of the VEGF receptor (VEGFR-2) for HRPC co-cultured with HUVEC. Hypoxia treatment significantly enhanced (8- to 10-fold higher than normoxia controls) VEGF secretion into media whether cells were cultured alone or in a co-culture. Also, hypoxia was found to result in a 3- and 2-fold increase in NFκB and HIF-1α mRNA expression by HRPC and a 4- and 6-fold increase in NFκB and HIF-1α protein by co-cultures, whether non-contacting or contacting.

Treatment of HRPC cells with hypoxic HUVEC-CM activated and promoted the translocation of NFκB and HIF-1α to the nuclear compartment. This finding was subsequently confirmed by finding that hypoxic HUVEC-CM resulted in higher expression of NFκB and HIF-1α in the nuclear fraction of HRPC and corresponding decrease in cytoplasmic NFκB and HIF-1α. Lastly, hypoxic conditioned media induced a greater formation of capillary-like structures (angiogenic response) compared to control conditioned media. This effect was attenuated by exogenous anti-human VEGF antibody, suggesting that VEGF was the primary factor in the hypoxic conditioned media responsible for the angiogenic response.

**Conclusions:**

These findings suggest that intercellular communications between HRPC and HUVEC lead to the modulation of expression of transcription factors associated with the production of pro-angiogenic factors under hypoxic conditions, which are necessary for an enhanced neovascular response. Our data suggest that the hypoxia treatment results in the up-regulation of both mRNA and protein expression for VEGF and VEGFR-2 through the translocation of NFκB and HIF-1α into the nucleus, and results in enhanced HRPC-induced neovascularization. Hence, a better understanding of the underlying mechanism for these interactions might open perspectives for future retinal neovascularization therapy.

## Background

Neovascularization (angiogenesis) is defined as the formation of new blood vessels by sprouting of endothelial cells from pre-existing vessels. This is a multistep process, which is controlled by opposing regulatory factors and involves endothelial cells migration, proliferation, degradation of the underlying basement membrane, and assembly into tubes [1,2]. Neovascularization plays a crucial role in several ocular diseases, including age-related macular degeneration, retinopathy of prematurity and proliferative diabetic retinopathy, which are major causes of blindness [3-6]. It often results in vitreous hemorrhage, retinal detachment, neovascularization glaucoma and subsequent vision loss. It is believed that tissue damage can stimulate release of angiogenic factors resulting in capillary proliferation [7-9]. Neovascularization is also essential for tissue repair, fetal development, and the female reproductive cycle. Changes in the regulatory factors, e.g., VEGF, may be directly related to pathological retinal diseases. These mediators can stimulate neovascularization directly by interacting with receptors on the endothelial cell surface, or indirectly by attracting and activating accessory cells.

Hypoxia is considered to be one of the key factors triggering angiogenesis by inducing angiogenic factors (like VEGF) and their receptors [10-12]. A number of studies [13-15] have shown that hypoxia plays a major role in triggering ocular neovascularization by inducing several angiogenic factors (e.g., vascular endothelia growth factors (VEGF) fibroblast growth factor (FGF), platelet-derived growth factor, (PDGF) and several others. VEGF is a 45 kDa glycoprotein and six isoforms of human VEGF have been identified that differ in their tissue specific expression pattern as well as in their biochemical and biological properties. VEGF as an angiogenic factor, multiple downstream signaling pathways have been implicated in the modulation of VEGF-dependent effects, including the regulation of cell survival, gene expression, cell proliferation and cell migration [16,17]. However, all isoforms will bind the two VEGF receptors [18,19]. Both receptors, VEGF receptor-1 (VEGFR-1 or Flt-1) and VEGFR-2 (or Flk-1/KDR) exhibit tyrosine-kinase activity (RTK).

Adaptation to hypoxia involves appropriate alterations in the expression of a number of angiogenesis-responsive genes. Hypoxia-induced genes are regulated by a critical transcriptional factor, namely hypoxia inducible factor-1 (HIF-1α) [11]. HIF-1α is a widely expressed heterodimeric protein composed of HIF-1α and ARNT subunits, both of which belong to the basic helix-loop-helix PAS family [20]. HIF-1α is a heterodimeric transcriptional factor that activates the transcription of several genes in response to low oxygen tension, including erythropoietin, transferrin, inducible nitric oxide synthase and VEGF. HIF-1α binds to DNA motifs hypoxia response elements (HREs) which have been found in the isoforms of a variety of genes that are overexpressed during neovascularization. Another factor, nuclear factor kappa B (NFκB) regulates the transcription of many genes required for neovascularization, cells adhesion, differentiation, proliferation and apoptosis [21-23]. Hypoxia can activate NFκB, which in turn, regulates the multiple human pathologies, including retinal neovascularization, diabetic retinopathy, inflammatory diseases, atherosclerosis and cancer. Hypoxia-mediated NFκB activation initiates a pathway that involves phosphorylation and subsequent degradation of the NFκB inhibitor (IKB) resulting in the cytoplasmic release and nuclear translocation of NFκB.

Many angiogenic factors are also implicated in neovascularization in the eye, where the source could be retina, pericytes, EC, or RPE. It has been suggested that an imbalance of growth factors may have a pathogenic role in retinal neovascularization. Additionally, hypoxia has been shown to induce the expression of VEGF in matured retinal cells [24-29]. To the best of our knowledge, this is the first study with HRPC co-cultured with HUVEC to examine as a model of retinal neovascularization. Our use of the HRPC cells as a model of retinal neovascularization was based on the fact that HRPCs represent only about 0.2% of pigmented cells in the ciliary margin zone, and has been shown to be multipotent, proliferative and express the neuroectodermal marker nestin [25]. HRPC can differentiate into retinal neurons and Mûller glia, including photoreceptors [30]. The aim of this study was to elucidate the mechanism of interaction between HRPC and HUVEC that modulates a neovascularization response. We established a trans-well co-culture system of endothelial cells (HUVEC) and HRPC under normoxia and hypoxia conditioned, and evaluated the effect of these environments as well as conditioned media from normoxia or hypoxia obtained from both cells on the angiogenic response. Further, we also studied the role of HUVEC-CM on the translocation of the transcriptional factors NFκB and HIF-1α in the HRPC cells. We found that hypoxia can affect both HUVEC and HRPC cells' gene expression independently. The factors are released into the media and can modulate endothelial-retinal cell interactions, which may lead to a number of neovascularization disorders, including retinal diseases.

## Methods

### Cell Culture

#### Human retinal progenitor cells (HRPC) and umbilical vein endothelial cell (HUVEC) propagation

The human retinal progenitor cells (HRPC) were characterized by and obtained from Dr. Kamla Dutt [30-32]. Cells were maintained routinely in Dulbecco Modified Eagle Medium (DMEM): nutrient mixture F-12 (1:1); DMEM/F-12 (Invitrogen) supplemented with 10% fetal bovine serum, 2mM L-glutamine, 0.075% sodium bicarbonate. The cells cultured were maintained at 37°C in a humidified air/CO_2 _(95:5, v/v) atmosphere and used 42-45 passages. At confluence, the media were replaced with serum-free 24 h before; cells were used for co-culture or exposed to normoxia or hypoxia conditions.

Human umbilical vein endothelial cells (HUVEC) were obtained as primary cultures from Cambrex Biosciences (San Diego, CA). HUVEC (passages 3-6) were cultured routinely in EMG-2 Bullet kit composed of endothelial cell basal medium-2 (EBM-2 medium) supplemented with 2% fetal bovine serum, human VEGF, human epidermal growth factor (hEGF), insulin-like growth factor-1, and ascorbic acid, gentamicin sulfate hydrocortisone, and heparin, as described by the manufacturer protocol [33]. When HUVEC were cultured in co-culture with HRPC or exposed with hypoxia, cells were maintained in serum free EMG-2 medium without any growth factor for 24 h.

#### Co-culture of HRPC and HUVEC in transwell systems

**Figure 1:**
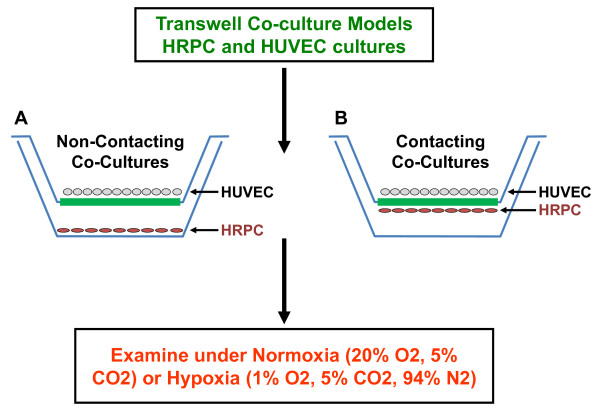
**Two different modes of transwell co-culture system**. (A) Non-contact: HRPC and HUVEC cells were co-cultured in two different compartments (insert membrane and well); cells may communicate through the pore of the membrane. (B) Contact: both cells may be cultured on the insert membrane thus allowing extensive and direct cell-cell interactions

Non-contacting and contacting co-culture transwell cell culture systems were developed to study the cross biological activity of HRPC and HUVEC. The non-contacting co-cultured cells (Figure 1A) were prepared as follows: HRPC cells were plated on the bottom of the six-well transwell cell culture system (Pore size 0.4 μm; Costar Corp. USA) using the complete media and culture environment as describe above. The HUVEC were cultured onto the membrane of transwell cell culture inserts and allowed to grow overnight using the above mentioned condition. The next day the cells were washed with the serum-free media and incubated for 24 h in serum-free medium without growth factors. After 24 h of growth in serum-free media, the HUVEC cultured on membrane transwell insert were placed into the six-well pates cultured containing the HRPC to initiate the experiment.

The HRPC and HUVEC cells are grown in the non-contacting co-culture system for 24 h, which allow bidirectional diffusion of soluble factors. Cultures were serum and growth factor deprived for 24 h before the normoxia/hypoxia environment studies were initiated. All cultures were 80-85% confluent by visual inspection on the day that hypoxia experiments were initiated. The second set of the cell-cell interactions were examined between HRPC and HUVEC using contacting co-culture model maintained in transwell plates (Figure 1B). Briefly, the HRPC were seeded on the bottom on transwell system membrane inserts and HUVEC were seeded on the top. All cultures were incubated at 37°C in a humidified air/CO_2 _(95:5, v/v) atmosphere for the duration of the experiment. The next day media were aspirated and replaced with fresh EMB medium without serum and growth factors for 24 h before the normoxia/hypoxia conditions. Finally, the transwell plates were placed under normoxia and hypoxia incubators for specified period of time.

### Hypoxia Treatments

Monolayer cultures were incubated in a hypoxic environment by the following protocol. Briefly, media were pre-equilibrated with a hypoxic gas mixture (1%O_2_, 5%CO_2_, and 94%N_2_). Cultures were replenished with the hypoxic medium, placed in the hypoxia incubator and maintained for 24 h to 48 h at 37°C. Control monolayer cultures were maintained in a normoxic environment, which refers to standard cell culture in a humidified incubator (20% O_2_, 5% CO_2_, and 75% N_2_). All cells maintained in normoxic condition showed no morphological changes by light microscopy. The trypan blue exclusion test of cell viability showed cells to be >98% viable, and could then be sub-cultured normally. Cells incubated under normoxia conditions from the same batch, and subsequently sub-cultured, were used as control.

### Preparation of HRPC/HUVEC conditioned medium (HRPC-CM/HUVEC-CM)

**Figure 2:**
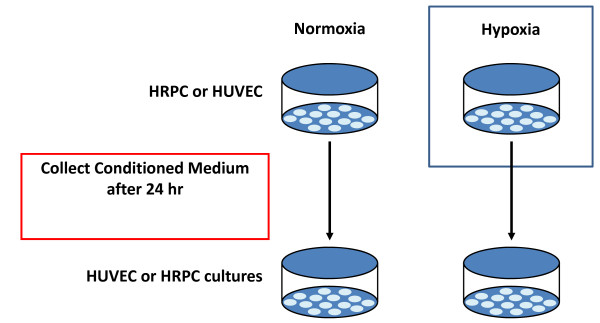
**Conditioned Medium collection**. Experimental protocol used in this study to collect the conditioned medium from normoxia/hypoxia environments. Collected conditioned medium were used to examine the effect of the factors released by HRPC on HUVEC and effect of the factors released by HUVEC on HRPC.

In a separate set of experiments, we evaluated the biological activity of conditioned medium (CM) obtained from HRPC (as HRPC-CM) and HUVEC (as HUVEC-CM) in normoxia and hypoxia condition [Figure 2]. HRPC cells were seeded in a 75 cm2 flasks with DMEM/F-12 with above mentioned supplements. HUVEC cells were cultured in EBM-2 medium with above mentioned supplements for 24 h. The cells were grown to 80% confluence then washed twice with the respective serum-free medium. Finally, the medium was replaced again with serum-free medium for another 24 h in normoxia and hypoxia condition. The CM from both cells under normoxia and hypoxia were collected, centrifuged at 1000 rpm for 10 min to remove cellular components and filtered through 0.2-μm filters (Millipore, Billerica, MA) before use. Medium from dishes under normoxic conditions was used as a control. The aliquots were stored at -80°C in a freezer.

### Quantifying secreted VEGF protein levels by ELISA

Changes in environmental factors in the cells culture can be differentially regulated by diverse signals. Therefore, we thought it was important to determine how hypoxia and co-culture interact in the regulation of VEGF secretion. Conditioned medium were collected, as described above, for the determination of secreted VEGF levels by ELISA. VEGF was assessed using commercially available human VEGF-ELISA kit (R&D Systems, Minneapolis, MN), performed according to manufacturer's instructions and normalized to protein content (BioRad, CA). The experiments were repeated three times in triplicates and are shown as mean ± SEM. The level of VEGF is expressed as pictograms of VEGF protein per milligram protein.

### Capillary-like Tube Formation Assays

The effects secreted factors from HRPC-CM under normoxia and hypoxia were examined in the formation of capillary tube-like structures by HUVEC. The matrigel assay is a frequently used method for detection of angiogenesis in vitro. Briefly, the growth factor reduced Matrigel (BD Biosciences, San Jose, CA) was added to pre-chilled 48 well culture plates and allowed to polymerize at 37° C for 2 h. HUVECs (4 × 10^5 ^cells/well) were plated in the Matrigel-coated plates and the cells cultured in HRPC-CM from normoxia and hypoxia conditioned for 24 h at 37°C with 5% CO_2_. To study the role of VEGF in capillary tube formation, parallel cultures were treated with 20 ng/ml neutralizing anti-VEGF antibody, which were added at the time of HUVEC seeding. VEGF (50 ng/ml) was used as the positive control. Angiogenesis (capillary cord formation) were evaluated after culturing for 24 and 48 h and resulting tube-like capillary structures were examined by using an Olympus TMS inverted phase contrast microscope. Photomicrographs were documented with an Olympus digital camera.

### Evaluating the expression of angiogenic protein by Western blot

The total cellular protein was isolated and used to examine the expression of angiogenic factors such as: VEGF, VEGF receptors (VEGFR-1 and VEGFR-2), and transcriptional factors (HIF-1α and NFκB). All these proteins play a major role in regulation of the retinal neovascularization. Briefly, after normoxia/hypoxia incubation for 24 h, media were collected and cells were washed with the PBS. The total proteins were extracted from the cell layers using M-PER Mammalian Protein Extraction Reagent (Pierce; CA) containing a protease inhibitor mixture (Sigma, CA). The protein extracts were centrifuged for 15 min at 15,000 rpm at 4° C and the supernatant were saved for further analysis. Total protein concentration of each sample was determined by the Bradford method as per manufacturer protocol (BioRad, CA) with bovine serum albumin as a standard. 30 μg total cellular proteins were analyzed on SDS/PAGE under reduced conditions. Before loading, all cell lysates were added to an equal volume of Laemmli sample buffer and heated at 95°C for 5 min, and samples were resolved by SDS-PAGE. The fractionated proteins were transferred to a polyvinylidene difluoride (PVDF) membrane. The membrane was blocked with 5% nonfat milk (BioRad, CA) in Tris buffered saline (TBS) with 0.05% Tween-20 (TBS-T) for 1 h at RT followed by incubation for 2 h with the primary antibodies in 2% milk in TBS-T. The membrane was washed three times with TBS-T for 10 min, and then incubated with the HRP-conjugated secondary antibodies, diluted in 2% milk in TBS-T, for 1 h, followed by three washes of 15 min in TBS-T. All membranes were probed with antibodies to β-actin to correct for loading and transfer differences among samples. The membranes were visualized by an enhanced chemiluminescence (ECL) Western Blotting Analysis System (Amersham).

### Evaluating the expression of angiogenic transcriptions by RT-PCR

Total RNA was extracted from cultures (control monolayer and co-cultured under normoxia and hypoxia conditions) using the extraction solution Triazol reagent (Invitrogen, CA) according to the supplier's instructions. The purity and concentration of RNA were quantitated by absorbance measurement at 260/280 nm. The quality of RNA was also checked by the agarose gel electrophoresis. Total RNA (1 μg from each sample) was run on 1% agarose gel, then stained with ethidium bromide, and examined the 28S and 18S rRNA bands on a UV transilluminator. No significant degradations were observed in any total RNA samples. The extracted RNA was then analyzed by RT-PCR for VEGF, VEGF receptor-2 (VEGFR-2), and transcriptional factors (NF_k_B and HIF-1α). The transcription of the housekeeping gene 18S RNA was used as internal controls (Ambion Inc. TX). The list of primers for RT-PCR to provided in Table 1. The reverse transcriptase (RT) reaction was performed with 1 μg of RNA, random hexamers, and Superscript II reverse transcriptase (Invitrogen, CA), according to the manufacturer's instructions. RT-PCR was conducted using the Gene Amp PCR System 2400 (Applied Biosystems) under the conditions described in the figure legends. The reaction mixture was first denatured at 95°C for 10 min, and the PCR conditions applied for 30 cycles. The amplified products were analyzed by electrophoresis on 1.8% agarose gels, and the ethidium bromide stained bands were visualized by UV transillumination. All images were saved in TIFF format. The levels of expression were quantitated by densitometer scanning. The target PCR products were analyzed by determining the intensity of band by gel-pro software (USA) which is a PC version of the image analysis software.

**Table 1:** List of Primers:

VEGF	5'-GCACCCATGGCAGAAGGAGGAGG-3' 5'-TTCCCGAAACCCTGAGGGAGGCT-3'
**VEGFR2**	5'-TTGTGACCCAAGAATGTGTCTGTG-3' 5-'CGAACTCTACTTTAGCCCAACTCG-3'

**NFκB**	5'- GCC ATG GAC GAA CTG TTC CCC-3' 5'- GCC ATG GAC GAA CTG TTC CCC-3'

**HIF-1α**	5'-GTCGGACAGCCTCACCAAACAGAGC-3' 5'-GTTAACTTGATCCAAAGCTCTGAG-3'

**18S RNA**	QuantumRNA Classic II (Ambion Inc. TX)

### Expression and Translocation of NFκB and HIF-1α

We also examined the expression and translocation of NFκB and HIF-1α in HRPC cultured in HUVEC-CM (normoxia/hypoxia) by western blot analysis and immunofluorescence studies.

#### Immunofluorescence studies

HRPC cells were cultured on 4-well chamber slides to approximately 80% confluence; then the cells were grown overnight under serum free medium. The next day, the medium was removed and cells were further cultured with HUVEC-CM (normoxia and hypoxia) at 37^o ^C in a humidified air/CO_2 _(95:5, v/v) atmosphere for 24 h. Cultures were then rinsed with pre-warmed PBS and fixed with 4% paraformaldehyde (Sigma) in PBS for 10 min at room temperature. The fixed cultures were washed and permeabilized with a solution of PBS containing 0.1% Triton X-100 (sigma) in PBS. Non-specific antibody binding sites on the cells were blocked by using 10% goat serum for 1 h at room temperature before incubation with primary antibodies. Cultures were then incubated with primary antibody rabbit polyclonal antibody against NFκB and HIF-1α (Santa Cruz Biotechnology, CA) overnight at 4° C. Cells were washed and incubated with FITC-conjugated anti-rabbit IgG (1:100 dilution) for 1 h at room temperature. For nuclear counter-stain DAPI (Molecular probe; CA) was used as per manufacturer's protocol.

#### Protein translocation

Cultures were incubated as described above for culture of normoxia/hypoxia HUVEC-CM-treated cells. After incubations, the medium was removed and cultures were washed with PBS. Cytoplasmic and nuclear proteins for Western blots were prepared using Nuclear Extract kits (Active Motif, Carlsbad, CA) according to manufacturer's suggested protocols. Protein concentration was determined by the Bradford method (Bio-Rad, Richmond, CA). Translocation of NFκB and HIF-1α were determined by western blotting as described above.

## Results

### HRPC-CM induces neovascular responses by HUVEC

**Figure 3:**
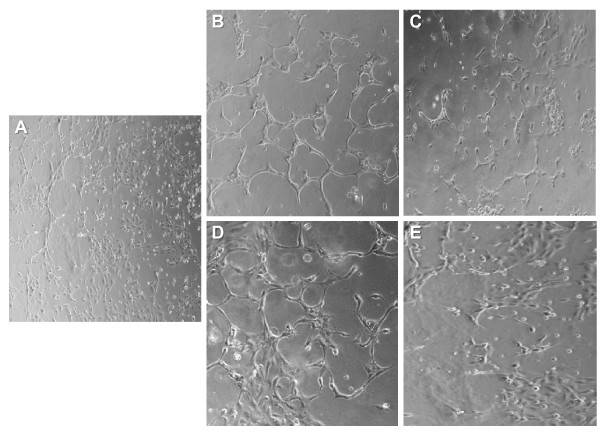
**Effect of normoxia and hypoxia HRPC-CM on capillary tube formation in the HUVEC**. Formation of capillary tubes by HUVECs in matrigel was evaluated in a condition medium obtained from normoxia and hypoxia HRPC-CM (see Material and Methods section). Representative photographs of Matrigel-coated cultured HUVEC cells after incubation with A) in serum-free medium, B) in HRPC-CM (normoxia), C) in HRPC-CM (normoxia) with anti human VEGF antibody; D) in HRPC-CM (hypoxia), E) in HRPC-CM (hypoxia) with anti human VEGF antibody. When HUVEC exposed to the basal medium or normoxia HRPC-CM, they were minimally spread out (A, B). In contrast, HUVEC were exposed to the hypoxia HRPC-CM within 24 hr assembles into capillary tube structures (D). When VEGF neutralizing antibodies were added to the conditioned medium, the tube formation was attenuated (C, E). Representative photomicrographs (20X magnification) are shown. Data shown are representative of three experiments done in triplicate.

The effect of HRPC-CM from hypoxia or normoxia treated cells on the induction of capillary-like structures (angiogenesis or neovascularization) in vitro was investigated using a well-established model - the matrigel in vitro angiogenesis assay. Control HUVEC (Figure 3A) cultures were grown on matrigel-coated plates for 24 h in serum free conditions. Treatment of HUVEC with normoxic HRPC-CM showed very weak neovascular response (Figure 3B) although somewhat blunted compared to that induced by hypoxic HRPC-CM (Figure 3D). This effect was also attenuated by the simultaneous treatment with anti-VEGF antibodies (Figure 3C). On the other hand, HUVEC treated with hypoxic HRPC-CM for 24 h was found to have the highest neovascular response (Figure 3D) which was attenuated by the simultaneous treatment with anti-VEGF antibodies (Figure 3E). This suggests that the primary factor in the HRPC-CM responsible for the neovascular response may be VEGF.

### VEGF secretion by individual cultures and co-cultures

**Figure 4:**
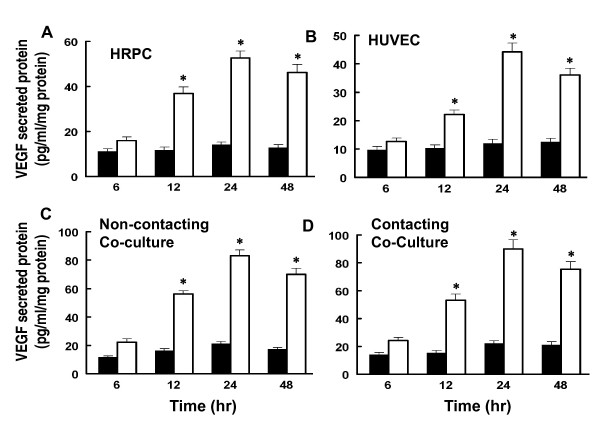
**The effects of hypoxia and co-cultured of HRPC and HUVEC on VEGF secretion**. Both HRPC and HUVEC were cultured singly and as co-cultures under normoxia (■) and hypoxia (□) conditions for specified time and medium were collected as described in Material and Methods. The secreted VEGF was determined by ELISA as described in above. The values presented as bar graphs give the means ± S.E.M. of quadruplicate from three independent experiments. The effects of both cells cultured/co-cultured on the secretion of endogenous VEGF protein are statistically significant under hypoxia conditions. Data shown is mean ± S.E.M. of three independent experiments (n = 4), *P>0.001.

We measured the levels of VEGF released into the culture media of individual cultures of HRPC and HUVEC as well as co-cultures of these cell lines, following cultures under hypoxic conditions for 6, 12, 24 and 48 h. Control cultures were treated the same except they were maintained in normoxic conditions. Co-cultures were setup as shown in Figure 1. Media from the various cultures were collected and analyzed for the levels of total VEGF by ELISA. We found that hypoxia clearly enhanced VEGF secretion by all the cultures (Figure 4). For all cultures, the VEGF level in the media shows a time-dependent increase from 6 to 48 hours. At the 24 h time point, there was a five-fold increase in VEGF level for HRPC (Figure 4A) and HUVEC (Figure 4B). Our co-culture data showed an 8- to 10-fold increase for the non-contacting or contacting co-cultures (Figure 4C & D) for hypoxia treated cultures compared to normoxia controls. There was no significant difference noted for VEGF secretion by cells in co-culture with contacting or non-contacting between the two cell lines.

### Effect of hypoxia on VEGF and VEGFR-2 expression

**Figure 5:**
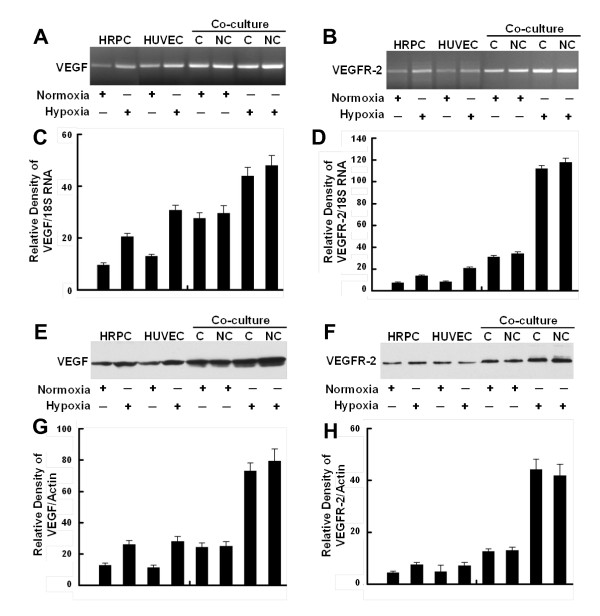
**Confirmation of gene expression**. Representative RT-PCR showing the expression of mRNAs for VEGF (A) and VEGFR-2 (B) in HRPC, HUVEC cultured alone and HRPC co-cultured with HUVEC under normoxia and hypoxia conditioned for 24 h. Densitometric analysis of mRNA expression of VEGF (C) and VEGFR-2 (D) presented as a ratio of VEGF and VEGFR-2 mRNA over 18S RNA. Representative Western blot analysis using VEGF (E) and VEGFR-2 (F) antibodies confirm the expression levels of these proteins. VEGF (homodimer, molecular mass ~ 44kDa) and VEGFR-2 (homodimer, molecular mass ~ 220 kDa) were expressed on HRPC and HUVEC cells. A β-actin antibody was used to normalize densitometer tracing values for differences in loading and the transfer efficiencies. Both of the experiments were repeated three times with similar results. Figure (G, H) bar graphs of quantitative analysis of Western blot data presenting calculated means +/- S.E. of densitometer tracings values from three independent experiments.

We also examined the transcriptional expression of VEGF and VEGFR-2 as well as the expressions VEGF and VEGFR-2 protein by cultures maintained in hypoxia for 24 hr. Figure 5A and 5B show that hypoxia treatment of either HRPC or HUVEC cultured alone resulted in a 2- to 2.5-fold higher expression of VEGF and VEGFR-2 mRNA. However, hypoxia was able to induce about a six-fold higher expression of VEGF and a four-fold higher expression of VEGFR-2 by co-cultures of HUVEC and HRPC. There were no significant differences noted for VEGF and VEGFR-2 expression when the two cell lines were co-cultured with contacting or non-contacting set-ups. These findings for co-cultures appear to be an additive effect of the responses of the individual cell lines.

We also observed that hypoxia induced an increase in VEGF and VEGFR-2 protein expression following western blot analysis [Figure 5E and 5F]. Our Western blot data support mRNA data with the low protein expression levels of VEGF and VEGFR-2 in HRPC and HUVEC were cultured under normoxia environment. We have found that exogenous VEGF expression is increased in HRPC and HUVEC cultured alone under hypoxia condition as compared with normoxia. There was a four-fold increase in VEGF expression by HRPC exposed to hypoxia for 24 h, as compared with normoxia. When HRPC were cultured in presence of HUVEC under hypoxia, there was a further two-fold increase in VEGF expression, compared to HRPC cultured alone under hypoxic conditions. In addition, our data indicated that hypoxia was capable of inducing not only VEGF expression, but also its receptor - VEGFR-2 when HRPC were co-cultured with HUVEC for 24 h. Data from RT-PCR and Western blot analysis are consistent with our VEGF ELISA results, and showed an increased in VEGF in all the hypoxia treatments and co-cultures compared with normoxia controls. These results suggest the association of VEGF-VEGFR-2 signaling regulate the retinal neovascularization.

### Role of hypoxia on the expression of NFκB and HIF-1α

**Figure 6:**
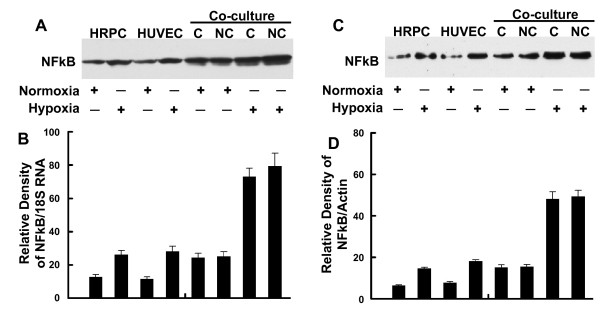
**RT-PCR and Western blot analysis of NFκB**. Total RNA and total protein were extracted from HRPC and HUVEC cultured alone or co-cultured under normoxia and hypoxia conditioned for 24 h. The expression of NFκB was measured by (A) electrophoresis of RT-PCR, (C) Western blot analysis in the HRPC and HUVEC. Figures (B, D) the band intensities corresponding to the NFκB were quantified by image analysis using a Bio-Rad scanning densitometer and Quantity One analysis software. Data were shown as ratio of NFκB densities to that of 18S RNA for RT-PCR and β-actin antibody was used to normalize Western blot for differences in loading and the transfer efficiencies. All data were expressed as mean +/- SE and results are representatives of three independent experiments.

Cultures of HRPC and HUVEC (alone or as co-cultures), maintained under normoxia (control) and hypoxia, were harvested, then total RNA and protein extracted. These extracts were used to measure the expression of transcription factors NFκB and HIF-1α. Figures 6A shows that either cell line alone constitutively expressed low levels of NFκB mRNA under normoxia conditioned. Hypoxia treatment of either HRPC or HUVEC resulted in a three- and two-fold increase in NFκB mRNA expression compared with normoxia controls, respectively. Additionally, we found that under control (normoxia) conditions, HRPC co-cultured with HUVEC as either contacting or non-contacting cultures, showed significantly higher levels of NFκB compared to single-cell cultures maintained similarly. In contrast, hypoxia treatment resulted in a much higher level of expression for NFκB (5-fold), compared to control co-cultures. There was more than a seven-fold induction of NFκB seen for co-cultures under hypoxia, compared to control co-cultures. To confirm our RT-PCR results, we also measured the protein expression for these transcriptional factors using western blotting. Total cellular proteins were isolated from culture cells maintained under hypoxia and normoxia (controls) for 24 h. Figure 6C shows western blots for NFκB; we found a 2.6-fold increase in NFκB expression by HRPC exposed to hypoxia for 24 h, compared with normoxia controls. A similar observation was found for hypoxia-treated HUVEC; there was a three-fold increase in NFκB expression. Lastly, we found that hypoxia was capable of significantly increasing the levels of NFκB expression by four-fold for co-cultures, whether non-contacting or contacting.

**Figure 7:**
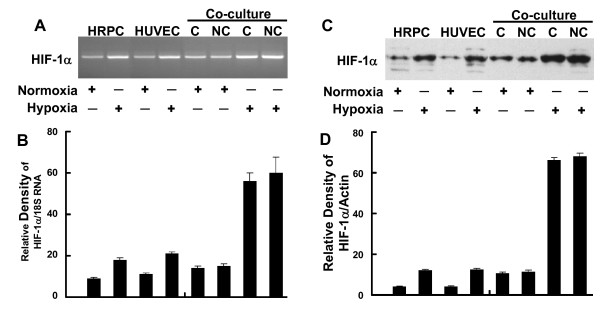
**RT-PCR and Western blot analysis of HIF-1α**. Total RNA and total protein were extracted from HRPC and HUVEC cultured alone or co-cultured under normoxia and hypoxia conditioned for 24 h. The expression of HIF-1α was measured by (A) electrophoresis of RT-PCR, (C) Western blot analysis in the HRPC and HUVEC. Figures (B, D) the band intensities corresponding to the HIF-1α were quantified by image analysis using a Bio-Rad scanning densitometer and Quantity One analysis software. Data were shown as ratio of HIF-1α densities to that of 18S RNA for RT-PCR and β-actin antibody was used to normalize Western blot for differences in loading and the transfer efficiencies. All data were expressed as mean +/- SE of three independent experiments.

Figure 7 showed the effects of hypoxia treatment on HIF-1α expression for single and co-cultured cells. Similar to the results found for NFκB, HIF-1α mRNA expression showed a two-fold increase in following hypoxia treatment for both HRPC and HUVEC cultured alone (Figure 7A). We also found that co-culture of these two cell lines resulted in a two-fold higher level of HIF-1α mRNA compared to single-cell HRPC cultures maintained similarly. Hypoxia treatment induced a four-fold increase in HIF-1α expression by co-cultures, compared to normoxia control co-cultures. Figure 7C shows that hypoxia resulted in a three-fold increase in HIF-1α protein by either HRPC or HUVEC cultured alone, but increased HIF-1α expression by six-fold for co-cultures. Contacting or non-contacting co-cultures had similar responses.

### Effect of HUVEC-CM on the Subcellular Localization of NFκB and HIF-1α

**Figure 8:**
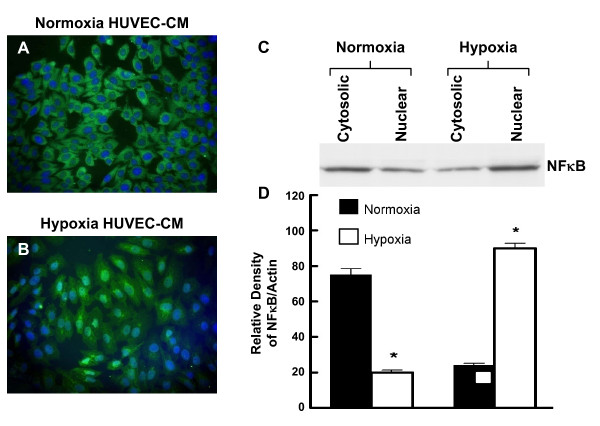
**Nuclear translocation of NFκB in HRPC**. The HRPC cells were cultured with normoxia/hypoxia HUVEC-CM for 24 hr and subcellular localization were done with immunofluorence and Western blot study. (A) HRPC cells were cultured with normoxia HUVEC-CM and intracellular location of NFκB was determined by immunofluorescence using primary anti-NFκB antibody and secondary antibody FITC labeled IgG. Nuclei were counterstained with DAPI. The immunofluorescence images observed in higher magnification showed that NFκB was more localized to the cytoplasm and less to the nuclei of the HRPC cells. (B) HRPC cells were cultured with hypoxia HUVEC-CM and labeled with anti-NFκB and nucleus staining with DAPI (blue). Hypoxia HUVEC-CM activates NFκB and translocated the most of the NFκB protein in the nuclear region of the HRPC cells. (C) Protein were extracted from the nucleus and the cytoplamic fraction of the HRPC cells after the cells were exposed with normoxia/hypoxia HUVEC-CM for 24 hr. 20 μg of protein from nuclear extracts and cytosol were separated by SDS-PAGE followed by western blotting with primary anti-NFκB antibody and followed by the secondary antibody HRP-conjugated. (D) Densitometry quantification of signal intensities of NFκB and β-actin was performed using a Bio-Rad scanning densitometer and Quantity One analysis software. Results are in arbitrary units and expressed as mean +/- SE of three independent experiments.

When HRPC single-cells cultures were treated for 24 h with either HUVEC-CM collected under normoxia (control) or hypoxia conditions, we observed significant effects on possible nuclear translocation of NFκB and HIF-1α. Figure 8A and 8B show immunofluorescent photomicrographs of HRPC treated with normoxic or hypoxic HUVEC-CM, respectively. With normoxic HUVEC-CM, there was higher NFκB immunofluorescence observed in the cytoplasm. This finding was subsequently confirmed by measuring the expression of NFκB protein in cytosolic and nuclear extracts using western blotting analyses (Figure 8C). Treatment with hypoxic HUVEC-CM resulted in the activation of NFκB and promoted its translocation to the nuclear compartment (Figure 8B) and resulted in higher expression of NFκB in the nuclear fraction of HRPC and corresponding decrease in cytoplasmic NFκB (Figure 8D).

**Figure 9:**
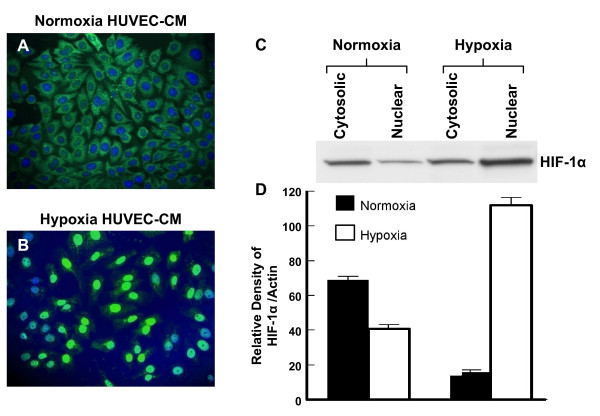
**The effect of hypoxia HRPC-CM on level and distribution of HIF-1α**. (A) HRPC cells were cultured with normoxia HUVEC-CM (B) HRPC cells were cultured with hypoxia HUVEC-CM. Cells were analyzed by immunofluorescence with primary antibody that recognize the HIF-1α and followed by secondary antibody FITC- conjugated IgG. Cells were counterstained with DAPI (blue) to label nuclei and analyzed using fluorescence microscopy. (C) Western blots analysis of HIF-1α in cytoplasmic and nuclear fractions were obtained from the HRPC cells were cultured with normoxia/hypoxia HUVEC-CM. (D) Densitometry quantification of signal intensities of HIF-1α and β-actin. Results are representatives of three independent experiments.

A similar finding was seen for the subcellular localization of HIF-1α (Figure 9). HRPC treated with the control normoxic HUVEC-CM showed HIF-1α was localized primarily in the cytoplasm (Figure 9A). Subsequently, treatment with hypoxic HUVEC-CM resulted in the translocation of HIF-1α to the nucleus (Figure 9B). Western blotting of proteins extracts from the cytoplasmic and nuclear fraction also showed a similar change. Treatment with hypoxic HUVEC-CM resulted in a higher expression of HIF-1α in the nuclear fraction compared to control cultures treated with normoxic HUVEC-CM (Figure 9C and 9D). Thus, these studies suggest that the hypoxia HUVEC-CM promoted the translocation of both NFκB and HIF-1α to the nuclear compartment of HRPC.

## Discussion

Retinal neovascularization is a complex process that is not completely understood. In retinal neovascularization, there are abnormally formed blood vessels and fibrous tissue that grow from the retina or the optic disk along the posterior surface of the vitreous. These retinal capillaries do not normally invade the vitreous or extend beyond the inner plexiform layer of the retina. However, in retinal diseases, e.g., diabetic retinopathy, the disruption of the balance between angiogenic and anti-angiogenic factors favors neovascularization, which enters the sub-retinal space, where leakage and bleeding lead to retinal detachment and photoreceptor death [25]. Clearly, the process of retinal neovascularization is mediated by the regulation of several angiogenic inducers, inhibitors, their respective receptors, and transcriptional factors. VEGF is a potent angiogenic and permeability enhancing factor causally linked to neovascularization in cancer, wound healing, diabetic retinopathy, and age-related macular degeneration [14,34]. Several reports have shown that VEGF is produced by several cell types within the eye (retinal pigment epithelial cells, glial cells, retinal capillary pericytes, endothelial cells, Müller cells and ganglion cells) [ 35 ] and play a role in the development of retinal neovascularization [13,36,7]. Aiello et al. [37] showed that VEGF played a significant role in mediating retinal neovascularization in diabetic patients. Using animal models of ischemic retinopathy and iris neovascularization, several researchers found that VEGF expression is upregulated in the retina during neovascularization [13,7,8,38]. Furthermore, inhibiting VEGF using VEGF receptor chimeric proteins, neutralizing antibodies, and antisense oligonucleotides in these animal models could reduce both retinal and iris neovascularization. Finding increased VEGF levels during retinal neovascularization has resulted in a controversy as to whether VEGF itself is sufficient to stimulate retinal neovascularization.

Increase VEGF expression could be the result of the retina microenvironment (hypoxia or ischemia) as well as changes in other factors such as pigment epithelium-derived factor (PEDF). The balance between the expression of VEGF and pigment epithelium-derived factor (PEDF) in modulating retinal neovascularization was investigated by Zhang and co-workers [39]. These researchers found a reciprocal interaction between VEGF and PEDF in retina; significant increases in VEGF expression was observed following silencing of the PEDF gene. Hypoxia has been shown to upregulate VEGF mRNA in retinal endothelial cells, pericytes, RPE cells, Muller cells and ganglion cells [14,40]. Our studies show that both HUVEC and HRPC express basal levels of VEGF and VEGFR-2 mRNA and protein, which was significantly increased by hypoxia treatment. Additionally, we found that co-cultures of these cell lines resulted in a synergistic increase in the expression of both VEGF and VEGFR-2, similar to that induced in single cultures by hypoxia. In fact, co-cultures treated with hypoxia showed a pronounced increase in the expression of both VEGF and VEGFR-2. However, contacting co-culture did not show a difference in response to hypoxia treatment from that of non-contacting co-cultures. Recently, Dardix and coworkers [41] also investigated the effects of co-culture (with retina pigmented epithelial cells - RPE) and hypoxia on endothelial cell expression of VEGF. These researchers reported that contact co-cultures upregulated both VEGF mRNA and protein expression to the same degree as hypoxia alone. In contrast to our findings, these researchers found that non-contacting co-cultures did not show an effect. However, these researchers used RPE cells for co-cultures as well as utilized CoCl_2_, a hypoxia mimetic to study the effect of hypoxia. In our studies we used human retinal progenitor cells (HRPC) cells for co-cultures; these cells express and secrete VEGF, among other soluble factors those have direct effects on endothelial cells [33]. In addition, we also used hypoxic culture conditions rather than the hypoxia mimetic, which may activate additional mechanisms not seen with the hypoxia mimetic.

Hence, our studies are consistent with the idea that hypoxia-inducing VEGF expression will lead to an enhanced neovascular response. These data not only highlight the important angiogenic properties of VEGF, but they also demonstrate the autocrine ability of VEGF to regulate the synthesis of multiple angiogenic proteins [42]. The autocrine effect of VEGF on retinal elements previously has been reported, for both retinal glial cells [43,44] and RPE cells [26], but not for the HRPC. To our knowledge, however, the regulation of VEGF receptor expression has not yet been investigated in retinal neovascularization induced by hypoxia co-culture system. Several lines of evidence indicate that enhanced VEGF expression in response to hypoxia is due to transcriptional activation [45,46]. Furthermore, this is the first time HRPC cells have been shown to have VEGF receptors (VEGFR-2) and induced the expression after hypoxia treatment. If similar autocrine functions of VEGF were present in HRPC cells, this would explain our observation of increased expression of VEGF and VEGFR-2 receptor after hypoxia and co-cultured conditioned.

It is well-established that hypoxia will stimulate neovascularization through inducing HIF1α, which translocates into the nucleus, binds to the hypoxia-response element, and up-regulates the expression of VEGF and other angiogenic factors [24]. Several studies have shown that hypoxia induces VEGF expression in matured retinal cells, which may play a major role in development of many retinal diseases. Our data from nuclear staining pattern and the immunoblotting of nuclear extracts indicate that HIF-1α, present in the cytoplasm of HRPC treated with normoxia HUVEC-CM, did translocate to the nucleus when cells were treated with hypoxia HUVEC-CM. HIF-1 may also have a function in retina that is unrelated to hypoxia because the constitutive level of HIF-1 expression in adult normoxic retina is much higher than that found for heart and lung, where it was undetectable by immunoblot or immunohistochemistry. VEGF is also constitutively expressed in the retina and it is possible that this is driven by the constitutive expression of HIF-1.

Another mechanism that may be activated by hypoxia treatment is NF-κB, which has also been suggested to play an important role in the induction of retinal neovascularization [21,23]. In the present study, we found that hypoxia has increases expression of NFκB mRNA and protein in HRPC/HUVEC cultured and co-cultured conditions. The hypoxic sensitivity of the NF-κB pathway and a wide variety of *in vitro *studies have demonstrated that exposure of various cell types to hypoxia activates NF κB signaling pathways. There are several reports that NFκB was activated in retinal glial cells, vascular endothelial cells, pericytes, and macrophage/microglia, all of which participate in neovascular disorders such as diabetic retinopathy. In the retina, NF-κB is localized in sub-retinal membranes and in microvessels and is activated very early in the course of development of retinopathy in diabetes. The activation of NFKB in retinal pericytes is responsible for the hyperglycemia-induced accelerated loss of pericytes observed in diabetic retinopathy. An inhibitor of NF-κB, PDTC inhibited NF-KB activation and reduced retinal neovascularization without discernible toxicity in a mouse model [45,46]. It also revealed that NF-KB might provide a basis for a specific and effective therapeutic regimen for various diseases associated with undesired angiogenesis. Normally, NFκB is present in the cytoplasm as a complex with members of the IkB inhibitor family. The hypoxia-induced activation of NFκB could result in the degradation of IkB. This in turn exposes the nuclear localization signal, allowing NFκB to be transported into the nucleus. In this study, we investigated the effects of normoxia/hypoxia exposed HUVEC-CM on NFκB nuclear translocation. Our studies showed that the HRPC cells did not significantly affect the nuclear translocation of NFκB when the cells were exposed with normoxia HUVEC-CM. However, nuclear translocation of NFκB was induced by hypoxia HUVEC-CM. Since nuclear localization of NF-κB is thought to be equivalent to NF-κB activation, we have shown that NF-κB was activated in HRPC cells by hypoxia HUVEC-CM, which may have triggered the transcription of NF-κB dependent genes and regulated the expression retinal neovascularization related genes.

## Conclusions

Our findings suggest that direct or indirect communications between HRPC and HUVEC cells results in the expression and activation of transcription factors associated with enhanced expression of pro-angiogenic factors under hypoxic conditions. These mechanisms play necessary roles in producing an enhanced neovascular response. Furthermore, our data indicate that the hypoxia treatment results in the up-regulation of both mRNA and protein expression for VEGF, and VEGFR-2 through the translocation of NFκB and HIF-1α to the nucleus, resulting in enhance HRPC-induced neovascularization. Hence, these results have provided a better understanding of the underlying mechanism involved in hypoxia-induced retinal neovascularization, which may generate new perspectives for future retinal neovascularization therapy.

## List of Abbreviations used

HRPC: human retinal progenitor cells; HUVEC: human umbilical vein endothelial cells; VEGF: vascular endothelial growth factor; VEGFR-2: vascular endothelial growth factor receptor -2; CM: condition medium

## Competing interests

The authors declare that they have no competing interests.

## Authors' contributions

RK conceived the ideas and development of the project, performed experiments, analyzed data, produced figures and graph, and wrote the manuscripts; SHH contributed to writing the paper; RK contributed work to experiments; GS contributed to the writing and the development of the project.

All authors have read and approved the final manuscript.
