# Correction: Co-culture of Retinal and Endothelial Cells Results in the Modulation of Genes Critical to Retinal Neovascularization

**DOI:** 10.1186/2045-824X-4-6

**Published:** 2012-03-26

**Authors:** Ravindra Kumar, Sandra Harris-Hooker, Ritesh Kumar, Gary Sanford

**Affiliations:** 1 Department of Microbiology, Biochemistry and Immunology, Morehouse School of Medicine, 720 Westview Drive, S.W., Atlanta, Georgia 30310, USA; 2 Department of Pathology, Morehouse School of Medicine, 720 Westview Drive, S.W., Atlanta, Georgia 30310, USA; 3 Undergraduate student, Department of Chemistry and Biochemistry, Georgia Institute of Technology, 901 Atlantic Drive, Atlanta, Georgia 30322, USA

## Correction

Following publication of our article [1] it was noted that Figures five E and five G were the same as Figures six A and six B. Figure 1 in this correction article is the correct version of Figure six that should have been included in the original article [1]. We apologize for any inconvenience caused by this error.

**Figure 1:**
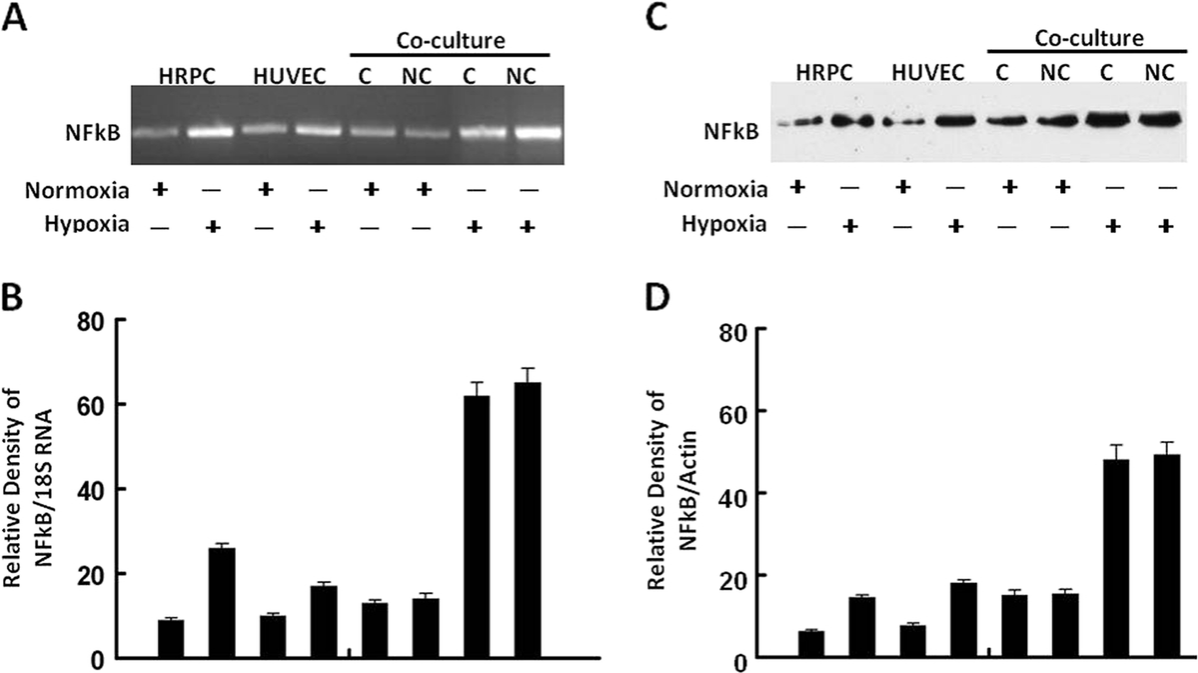
**RT-PCR and Western blot analysis of NFκB**. Total RNA and total protein were extracted from HRPC and HUVEC cultured alone or co-cultured under normoxia and hypoxia conditioned for 24 h. The expression of NFκB was measured by (A) electrophoresis of RT-PCR, (C) Western blot analysis in the HRPC and HUVEC. Figures (B, D) the band intensities corresponding to the NFκB were quantified by image analysis using a Bio-Rad scanning densitometer and Quantity One analysis software. Data were shown as ratio of NFκB densities to that of 18S RNA for RT-PCR and β-actin antibody was used to normalize Western blot for differences in loading and the transfer efficiencies. All data were expressed as mean +/- SE and results are representatives of three independent experiments.
